# 

**Published:** 1945

**Authors:** 


### The Bristol Medico-Chirurgical Journal

A JOURNAL OF THE MEDICAL SCIENCES FOR THE WEST OF ENGLAND AND SOUTH WALES

PUBLISHED UNDER THE AUSPICES OF

*THE BRISTOL MEDICO-CHIRURGICAL SOCIETY*

EDITOR

J. A. NIXON, C.M.G., M.D., F.R.C.P.

WITH WHOM ARE ASSOCIATED

A. E. ILES, O.B.E., M.B., B.S., F.R.C.S.

A. RENDLE SHORT, M.D., B.S., B.Sc., F.R.C.S.

W. MELVILLE CAPPER, M.B., B.S., F.R.C.S.

E. WATSON-WILLIAMS, M.C., Ch.M., F.R.C.S., Assistant Editor

---

"Scire est nescire, nisi id me

Scire alius sciret"

---

*VOL. LXII*

1945

BRISTOL: J. W. ARROWSMITH LTD.

### Contents of Vol. LXII.

| | Page |
|---|---|
| Gastroscopy: A Survey. By NORMAN C. TANNER, M.B., CH.B., F.R.C.S. .. | 1 |
| Acute Inversion of the Uterus Following Delivery. By CORALIE RENDLE-SHORT, M.B., Ch.M., M.R.C.O.G. .. .. .. .. .. .. | 9 |
| Observations on Some Rock Carvings of Surgical Instruments in East Africa. By M. NIERENSTEIN, D.Sc. .. .. .. .. .. .. .. | 14 |
| Selection of Medical Students. By FRANK BODMAN, M.D. .. .. .. | 18 |
| REVIEWS OF BOOKS .. .. .. .. .. .. .. .. .. | 22 |
| EDITORIAL NOTES .. .. .. .. .. .. .. .. .. | 27 |
| OBITUARIES .. .. .. .. .. .. .. .. .. .. | 31 |
| MEETINGS OF SOCIETIES .. .. .. .. .. .. .. .. | 37 |
| MEDICAL LIBRARY OF THE UNIVERSITY OF BRISTOL .. .. .. .. | 38 |
| THE ONE HUNDRED AND SEVENTY-NINTH LIST OF BOOKS, ETC. .. .. | 38 |
| PUBLICATIONS RECEIVED .. .. .. .. .. .. .. .. | 41 |
| LOCAL MEDICAL NOTES—EXAMINATION RESULTS .. .. .. .. | 42 |

420243

### The Medical Library of the University of Bristol

WITH WHICH IS INCORPORATED

**The Library of the Bristol Medico-Chirurgical Society**

---

*The following donations have been received since the publication of the list in the last issue.*

| | | |
|---|---|---|
| Geneıal Medical Council (1) .. .. .. .. | 3 | volumes |
| Miche Clarke Fund (2) .. .. .. .. | 19 | ,, |
| Professor C. Bruce Perry (3) .. .. .. | 2 | ,, |
| Royal College of Surgeons (4) .. .. .. | 1 | ,, |
| Royal Institute of Public Health and Hygiene (5).. | 1 | ,, |
| J. E. Shaw Fund (6) .. .. .. .. .. | 3 | ,, |
| Miss M. C. Shaw (7) .. .. .. .. .. | 2 | ,, |
| Dr. Kornel Terplan (per Professor Hewer) (8) .. | 1 | ,, |
| University of Otago Medical School (9) .. .. | 1 | ,, |
| Captain Richards (10) .. .. .. .. .. | 10 | ,, |

Unbound periodicals have also been received from Dr. S. B. Adams, Mrs. Hey Groves, Dr. T. Milling, Professor C. Bruce Perry, Dr. J. Odery Symes, U.S. Army Medical Corps, Messrs. John Wright & Sons, Ltd.

### THE ONE HUNDRED AND SEVENTY-NINTH LIST OF BOOKS.

The figures in round brackets refer to the figures after the names of the donors. The books to which no such figures have been attached have either been bought from the Library Fund or received through the *Journal.*

**Bailey, Hamilton** .. *Demonstrations of Physical Signs in Clinical Surgery* .. .. .. .. .. .. .. 9th Ed. 1944

**Bailey, Hamilton, and Bishop, W. J.** ..*Notable names in Medicine and Surgery* .. .. .. .. .. .. .. .. .. .. 1944

**Bailey, Hamilton (Ed.)** *Pye's Surgical Handicraft* .. .. .. 14th Ed. 1944

**Bailey, Hamilton (Ed.)** *Surgery of Modern Warfare,* 3rd Ed., 2 vols. (2) 1944

**Beaumont, G. C. and Dodds, E. C.** *Recent Advances in Medicine,* 11th Ed. (2) 1944

Best, C. H., and Taylor, N. B. *Physiological basis of Medical Practice* 3rd Ed. (2) 1943

Blacker, C. P. .. .. *Notes for the R.M.O. of an Infantry Unit* .. .. 1944

Bloor, W. R. .. .. *Biochemistry of the Fatty Acids and their Compounds, the Lipids* .. .. .. .. (2) 1944

Boyd, W. .. .. .. *A Textbook of Pathology* .. 4th Ed. (2) 1943

Broadbridge, H. G. .. *Murrell's What to do in Cases of Poisoning*..

Bunnell, Sterling .. .. *Surgery of the Hand* .. .. .. .. .. .. (6) 1945

Burroughs, G. F. .. *A Narrative of the Retreat of the British Army from Burgos* .. .. .. .. .. .. .. .. 1814

*Calendar of the Royal College of Surgeons of England* .. .. .. (4) 1944

Cecil, R. L. (Ed.) .. *A Textbook of Medicine* .. .. 6th Ed. (2) 1944

Cole, F. J. .. .. .. *History of Comparative Anatomy* .. .. .. 1944

*Collected Papers of the Mayo Clinic, Vols.* XXXIII—XXXV. .. .. ..

Coope, R. .. .. .. *Diseases of the Chest* .. .. .. .. .. .. (2) 1945

Crawford, A. M. .. *Materia Medica for Nurses* .. 5th Ed. 1942

Currie, J. R. and Mearns, A. G. *Hygiene* .. .. .. .. .. .. 2nd Ed. (3) 1945

Dible, J. H., and Daire, T. B. *Pathology* .. .. .. 2nd Ed. (2) 1945

Dilling, W. J. .. .. *The Pharinacology and Therapeutics of the Materia Medica* .. .. 18th Ed. (2) 1944

Durbeck, W. E. .. .. *The Impacted Lower Third Molar* .. .. (2) 1943

Fish, E. W. .. .. *Paradontal Disease* .. .. .. .. .. (2) 1944

Fisher, A. G. T. .. .. *Treatment by Manipulation* .. .. .. .. (2) 1944

Fulton, J. F... .. .. *Physiology of the Nervous System* 2nd Ed. (2) 1943

Gaddum, J. H. .. .. *Pharmacology* .. .. .. .. .. .. 2nd Ed. 1944

Gould, G. M. .. .. *Gould's Medical Dictionary* .. .. 5th Ed. 1943

Gunn, J. A. .. .. *Intro. to Pharmacology and Therapeutics*, 7th Ed. 1944

Harding, A. S. .. .. *A History of the Institute of Hygiene* .. (5) 1944

Illingworth and Dick .. *Textbook of Surgical Pathology* .. 3rd Ed. (10) 1944

Love, R. J. McNeill .. *A Guide to the Surgical Paper with questions and Answers* .. .. .. .. .. .. .. 2nd Ed. 1944

Mellor, J. W. .. .. *Modern Inorganic Chemistry* .. 9th Ed. (7) 1944

Minnitt, R. J. and Gillies, J. *Textbook of Anaesthesia* .. .. .. 6th Ed. 1945

Moore .. .. .. .. .. *Textbook of Pathology* .. .. .. .. .. .. .. 1945

Pannett, C. A. .. .. *Surgery* .. .. .. .. .. .. .. .. .. .. (2) 1944

Quick, A. J. .. .. *The Haemorrhagic Diseases and the Physiology of Haemostasis* .. .. .. .. .. .. .. .. (2) 1942

Rolleston, J. D. (Ed.) *British Encyclopædia of Medical Practice* :—
(*a*) *Cumulative Supplement* .. (6) 1945
(*b*) *Medical Progress*.. .. .. (6) 1945

Roxburgh, A. C. .. .. *Common Skin Diseases* .. .. 7th Ed. 1944

Sairll, T. D. .. .. .. *System of Clinical Medicine*.. .. 12th Ed. (2) 1945

Schmeidler, C. .. .. *Historical Survey of Pharmacy in Great Britain* (3) c1939

Starling .. .. .. .. *Principles of Human Physiology*.. 9th Ed. 1945

| | | |
|---|---|---|
| **Stead, G.** .. .. .. | *Elementary Physics* .. .. .. 6th Ed. (7) | 1942 |
| **Stockard, C. R. et al** .. | *The Genetic and Endocrinic Basis for Differences in Form and Behaviour* .. .. .. .. | 1941 |
| *Studies of Burns and Scalds* (M.R.C. Special Report 249).. .. .. .. .. | | 1944 |
| **Terplan, Kornel** .. | *Anatomical Studies on Human Tuberculosis*.. (8) | 1940 |
| **Tidy, H. L.** .. .. .. | *Synopsis of Medicine* .. .. .. 8th Ed. | 1945 |
| **Tobias, N.** .. .. .. | *Essentials of Dermatology* .. .. 2nd Ed. | 1941 |
| **Tylman, S. D.** .. .. | *Theory and Practice of Crown and Bridge Prothesis* .. .. .. .. .. .. .. .. (2) | 1944 |
| **Vedder** .. .. .. .. | *Medical Aspects of Chemical Warfare* .. (2) | 1944 |
| **Waller, H.** .. .. .. | *Clinical Studies in Lactation* .. .. .. (2) | 1938 |
| **Wampler, F. J.** .. .. | *Principals and Practice of Industrial Medicine* (2) | 1943 |
| **Wheeler, R. C.** .. .. | *Dental Anatomy and Physiology* .. .. (2) | 1944 |
| **Wolff, E.** .. .. .. | *Diseases of the Eye* .. .. .. .. Ed. 2 | 1942 |
| **Woodger, J. H.** .. .. | *Elementary Morphology and Physiology* 3rd Ed. | 1943 |
| **Young, J. H.** .. .. | *Caesarean Section* .. .. .. .. .. .. | 1944 |

### TRANSACTIONS, REPORTS, JOURNALS, ETC.

Annual Report of the Smithsonian Institute, 1943 .. .. .. .. ..

British Journal of Industrial Medicine .. .. .. .. .. .. .. Vol. I

Cancer Research .. .. .. .. .. .. .. .. .. .. .. .. .. Vol. I

Dentists Register, 1945 .. .. .. .. .. .. .. .. .. .. .. (1)

Hospitals Year Book, 1943—1944 .. .. .. .. .. .. .. .. .. ..

Medical Annual, 1944 .. .. .. .. .. .. .. .. .. .. .. ..

Medical Directory, 1945 .. .. .. .. .. .. .. .. .. .. .. ..

Medical Register, 1945.. .. .. .. .. .. .. .. .. .. .. .. (1)

Quarterly Cumulative Index Medicus, Vols. 35, 36, 1944 .. .. ..

Proceedings University of Otago Medical School, No. 22, 1945 .. (9)

Report of the Inter-Departmental Committee on Medical Schools (Goodenough Report), 1944 .. .. .. .. .. .. .. .. .. .. .. .. ..

Transactions of the Ophthalmological Society of the United Kingdom, Vol. LXIII, 1944 .. .. .. .. .. .. .. .. .. .. .. .. ..

### Publications Received

From GENERAL MEDICAL COUNCIL :

*Seventh Addendum to the British Pharmacopoeia,* 1932.

From WILLIAM HEINEMANN MEDICAL BOOKS LTD. :

*Outlines of Physical Methods in Medicine.* By G. D. KERSLEY, 1945. Price 6s.

*The Rheumatic Diseases.* By G. D. KERSLEY, M.D., and SIR FRANCIS FRASER, M.D. Second Edition, 1945. Price 15s.

From INSTITUTE FOR SCIENTIFIC TREATMENT OF DELINQUENCY :

*Psycho-pathology of Prostitution.* By E. GLOVER, 1945. Price 1s.

From MESSRS. H. K. LEWIS & CO. LTD.

*Cookery Book for Diabetics.* Issued by THE DIABETIC ASSOCIATION, 1945. Price 4s.

*The Infant—a Handbook of Management.* By W. J. PEARSON AND A. G. WATKINS, 1945. Price 4s.

From OXFORD UNIVERSITY PRESS :

*A Textbook of Psychiatry.* By HENDERSON AND R. G. GILLESPIE. Sixth Edition, 1944. Price 25s.

*Industrial Toxicology* (*Croonian Lecture,* 1942). By DONALD HUNTER, M.D., F.R.C.P., 1944. Price 10s.

From MESSRS. T. J. SMITH AND NEPHEW LTD. :

*Cellona Technique,* 1945.

From MESSRS. JOHN WRIGHT & SONS LTD. :

*Do you want to be a Nurse?* By E. DRYBROUGH-SMITH, 1945. Price 1s.

*Index of Differential Diagnosis of Main Symptoms.* By HERBERT FRENCH. M.D., F.R.C.P. Sixth Edition, 1945. Price 84s.

*Elements of Medical Treatment.* By SIR ROBERT HUTCHISON, M.D., F.R.C.P, Fourth Edition, 1945. Price 10s. 6d.

*Synopsis of Forensic Medicine and Toxicology.* By E. W. CARYL THOMAS, M.D. Second Edition, 1945. 10s.

*Groves' Synopsis of Surgery.* Edited by C. P. G. WAKELEY, F.R.C.S. Twelfth Edition, 1945. 25s.

*British Journal of Surgery.* Vol. XXXIII., No. 130, October, 1945.

### Local Medical Notes

The Edward Fawcett Memorial Prize and the Foyle Prize have both been awarded for 1945 to Mr. A. H. Levy.

The Hewer Pathological Prize for 1945 has been awarded to Mr. A. C. Edwards.

EXAMINATION RESULTS.

**University of Bristol.**—Students of the University have recently passed the following examinations :—

M.B., Ch.B.—*First Examination :* P. B. Bailey, Monica B. Bishop, H. J. Bland, S. H. Brown, Judith A. V. Davis, Dorothy R. Douglas, B. R. W. Evans, H. Haig, L. Harbin, Pamela J. Harris, Monica C. Haynes, R. Hill, P. M. Hunter, J. A. Jefferies, Megan D. M. Johnson, Helena B. Knottova, G. W. B. Peel, Barbara M. Philpott, G. A., Pilkington, J. F. Rowland, Rachel D. R. Sasieni, Mildred M. C. Shaw, L. P. Spence, D. T. I. Stuttaford, M. D. Turner, D. W. Wright, M. A. Feldman, H. I. Leigh, Hella R. Sadler, Joan H. Barnes, Ann H. Davies, R. D. Doyle, C. C. Ewing, Elizabeth M. Fraser, G. C. K. Herapath Marianne A. Taylor, Joan Walker. *In Organic Chemistry :* Rosalind P. Adams, R. M. Barnes, Jennifer M. Comely, Margaret J. Francis, Sybil Jacob, J. D. Sheward, June M. Smith, Margaret A. Tubbs.

M.B., Ch.B.—*Second Examination :* Barbara Brosnan, R. J. Carey, Suzanne K. R. Clarke, Anne Cousins, H. M. Dowling, C. C. Downie, D. N. Fitzgerald, Molly I. Govier, B. T. Hale, Pamela I. Hannaford, Kathleen J. Harrison, A. J. Lee, C. D. Linton, D. B. Peacock, Elizabeth Preston-Thomas, Jenny Pym, G. T. Salter, P. J. Speller, A. S. Wallace, M. P. Walker. (*Section I*) *:* Diana F. J. Duncan, Margaret Ford, Anne M. French, G. A. Hanks, Ruth E. Hazell, Joan A. Higginbottom, A. H. Levy, June Morgan, N. Sartori, B. F. Vaughan, Pamela L. C. Watson, Audrey M. Wells, R. H. Wood. (*Section II*) *:* Margaret J. Andrews, N. M. Gibbs, A. H. Laxton, J. S. R. R. Old, F. A. A. Ruggeri, G. D. Teague.

M.B., Ch.B.—*Final Examination* (*Section I*) *:* Grace V. Andrew, J. A. Ardis, W. E. Arnold, Kathleen Blott, Dorothy J. Blake, D. E. Bolt, Elizabeth Bryant, W. H. K. Carpenter, N. J. Cook, Margaret J. Cox, R. W. Drewer, (Mrs.) M. M. Fairband, Sheila E. Fraser, F. D. Kay, M. B. Lennard, (Mrs.) E. E. Napthine, J. H. K. Parker, Hazel M, Passmore, H. S. Paull, C. D. R. Pengelly, Dorothy E. M. Pierce. D. Purnell, Dorothy M. Reece, R. H. Scott, A. D. Stewart, T. Stratton,

Jean Tregear, (Mrs.) P. M. Valentine, Andre D. Verniquet, K. D. J. Vowles, Pamela Westhead, Kathleen G. Wright. (*Section II*) *:* J. A. Ardis, J. B. Brierley (1), Roma N. Chamberlain, W. P. Foster, (Mrs.) S. Gordon, Irene D. R. Gregory, A. W. Heaton-Ward, D. H. Johnson (1), (Mrs.) E. E. Napthine, W. Oldham, E. S. Short, Barbara F. Smith, Doris G. Virgo (1). *In Group I :* M. J. Cook, A. C. Edwards, J. R. Hawkings, R. A. J. Pearce, S. A. K. Stokes. *In Group II :* (Mrs.) I. M. M. Codd, (Mrs.) J. R. Oakes.†

M.B., CH.B.—*Final Examination :* M. Alms, Grace V. Andrews, R. W. Drewer, E. W. Emery, Margaret Lamb, Edith J. Lowick, J. D. Medhurst, Ruth M. Rocke. *In Group II :* N. J. Cook,†, A. C. Edwards,† J. G. R. Ellis, J. R. Hawkings,† R. A. J. Pearce,† S. A. K. Stokes.†

B.D.S.—*First Examination :* Claire Ascott, H. Maxwell, L. A. D. Tovey. *Supplementary :* D. Clifford, E. Farwell, P. P. Griffiths, L. M. Lloyd, A. R. Owen.

B.D.S.—*Second Examination :* A. J. P. Cousins, L. M. Lloyd, A. R. Owen, L. K. Walden.

B.D.S.—*Third Examination :* R. J. Brownlee, L. K. Walden. *In Dental Mechanics :* A. J. P. Cousins. *In Dental Mechanics, etc. :* P. J. Blyth,† W. Watson.†

B.D.S.—*Final Examination.—In Section I only :* J. M. Allen, G. A. J. Comyns (2, 3), A. N. R. Gibson, D. C. Sara, A. F. J. Smith (2). *In Section II* (*Dental Surgery*) *:* H. L. Leech,† I. A. Morgan,† Evelyn M. Pitt.†

L.D.S.—*First Examination :* J. T. Sanders. *Supplementary :* J. P. B. Pengelly.

L.D.S.—*Second Examination :* M. J. Bartlett, J. C. Fishpool, T. C. Hughes, Margaret J. Webb.

L.D.S.—*Third Examination :* J. C. Fishpool, T. C. Hughes.

L.D.S.—*Final Examination.—In Section I :* B. L. Evans, J. C. Nicholas, (Mrs.) M. P. Wood, J. F. V. Woolley. *In Section II :* T. W. Beer,† K. W. Bowrey,†, A. R. Brook,† C. Gibbs,† Eileen Rich,† L. H. Valentine,† J. F. V. Woolley.†

**University of Cambridge.**—M.B., CH.B.—*Final Examination :* J. M. Drew, G. H. Seale. *Part II :* J. H. E. Bergin,† W. R. Cole.†

**University of London.**—M.B., B.S.—*Final Examination :* Susanna Gordon.

**Royal College of Physicians.**—M.R.C.P.—J. F. Ackroyd.

**Royal College of Surgeons.**—F.R.C.S.—F. R. Hurford, P. B. Ryan.

**Royal College of Surgeons of Edinburgh.**—F.R.C.S. (Edin.).—D. C. Bodenham.

† Completing Examination.

**Royal College of Obstetrics and Gynaecology.**—M.R.C.O.G.—Dorothy M. Shotton, Dorothy J. Thompson.

**Conjoint Board.**—M.R.C.S., L.R.C.P.—J. A. Ardis (4, 6), J. H. Beatson (5), J. B. Brierley (4, 7, 8), Jyotikana Datta† (7, 8), J. M. Drew† (6, 7), J. G. R. Ellis† (4, 6, 7, 8), Pamela M. Farmer (5), B. L. Finzel† (7), W. P. Foster† (4, 6, 7, 8), R. G. Glen (5), B. T. Hale (9), J. R. Hawkins† (4, 6, 7, 8), Ruth E. Hazell (9), F. D. Kay (4, 6), J. D. Medhurst (4, 6, 7), Jean Musgrave (10), R. A. J. Pearce† (4, 6, 7, 8), G. H. Seale† (4, 6), W. Wagland (5).

L.D.S., R.C.S.—J. R. V. B. Gibson, G. E. Johns.

† Completing Examination.
(1) With Distinction in Forensic Medicine.
(2) With Distinction in Surgery.
(3) With Distinction in Pathology.
(4) Pathology.
(5) Pharmacy.
(6) Medicine.
(7) Surgery.
(8) Obstetrics.
(9) Anatomy.
(10) Physiology.

HONOURS.

D.S.O.—Lieut.-Colonel M. E. M. Herford, M.B., Ch.B., R.A.M.C.

C.B.E.—Brigadier G. S. Parkinson, D.S.O., R.A.M.C.

O.B.E.—Major A. E. Jowett, M.B., Ch.B., R.A.M.C.

Mentioned in Despatches.—Major-Gen. Sir Percy S. Tomlinson, K.C.B., D.S.O., A.M.S. Captain N. C. Coombes, R.A.M.C.

Bar to Military Cross.—Major P. K. Jenkins, M.C., M.B., Ch.B., R.A.M.C.

M.C.—Captain E. W. Moore, R.A.M.C.

D.F.C.—Flight-Lieutenant F. G. Daw, R.A.F.

ROLL OF HONOUR.

Killed in Action.—Sergt. J. A. Bentz, R.A.F., Sergt. M. G. Wood, R.A.F., Lieutenant A. R. B. McGillycuddy, S.L.I.

Wounded.—Major P. K. Jenkins, M.B., Ch.B., R.A.M.C., Captain C. J. R. Jacob, R.A.M.C., Captain E. W. Moore, R.A.M.C., Lieut. T. R. Stephens, R.A.M.C.

**Bristol Eye Dispensary.**—The Committee of the Bristol Eye Dispensary have arranged that patients may be seen by appointment. Application should be, if possible, by letter. Addressed " Appointments," Eye Dispensary, 17 Orchard Street, Bristol 1.

### Bristol Medico-Chirurgical Society

### President.

H. H. CARLETON, M.A., M.D. Oxon., F.R.C.P. Lond.

### President-Elect.

### Committee.

*Ex-Officio* { J. A. NIXON, *C.M.G.*, B.A., M.D. Cantab. (Editor of *Journal*).

(Vacant), (Hon. Librarian).

*Elected.*

C. CORFIELD, M.D. (Ex-President).
S. BRYAN ADAMS, Ph.D, M.B., Ch.B.
R. F. BARBOUR, M.A., M.R.C.P., D.P.M.
F. H. BODMAN, M.D.
BERYL D. CORNER, M.D.
T. F. HEWER, M.D.
G. D. KERSLEY, M.A., M.D., M.R.C.P.
G. L. ORMEROD, M.A., M.B., B.Chir.
H. K. V. SOLTAU, M.D.

### Honorary Secretary.

A. L. EYRE-BROOK, M.S.

### Honorary Treasurer.

FREDERICK SUTTON, M.C., M.D.

### University Library Sub-Committee.

A. R. SHORT, B.Sc., M.D. Lond., F.R.C.S.
C. BRUCE PERRY, M.D. Brist., F.R.C.P.
H. J. DREW SMYTHE, *M.C.*, M.S., M.D. Lond., F.R.C.S., F.R.C.O.G.

Medical Practitioners wishing to join the Society are requested to communicate with the Hon. Secretary, Mr. A. L. Eyre-Brook, Litfield House, Clifton Down, Bristol 8, The Annual Subscription is £1 1s., payable to Hon. Treasurer.

---

*Extracts from Journal By-laws.*

4.—The *Journal* shall be delivered free to Subscribers and also to Members of the Society whose subscriptions are not in arrear.

6.—The Annual Subscription to the *Journal* is 9/–, payable to Hon. Treasurer.

---

Communications intended for insertion in the *Journal* should be sent to the Editor, Dr. J. A. Nixon, 7 Lansdown Place, Clifton, Bristol.

Books for Review and Exchange Journals should be sent to the Assistant Editor, *Bristol Medico-Chirurgical Journal,* Medical Library, The University, Bristol. Reviews should be returned with the books to Mr. E. Watson-Williams, 65 Pembroke Road, Bristol 8.

Communications referring to the delivery of the *Journal* should be sent to the Hon. Treasurer, Dr. Frederick Sutton, 1 Pembroke Road, Bristol 8.

Volumes of the *Journal* can be bound upon application to J. W. Arrowsmith Ltd., Quay Street, Bristol, price on request. The inclusive Index for Volumes I—LI may be obtained, price 3/– (postage extra).

### Bristol Medico-Chirurgical Society

### PAST PRESIDENTS

1874—75. FREDERICK BRITTAN, M.D., B.S. Dub.
1875—76. ROBERT WILLIAM COE.
1876—77. SAMUEL MARTYN, M.D. Ed.
1877—78. AUGUSTIN PRICHARD, M.D. Berlin.
1878—79. HENRY EDWARD FRIPP, M.D. Würzburg.
1879—80. WILLIAM MICHELL CLARKE.
1880—81. JOSEPH GRIFFITHS SWAYNE, M.D. Lond.
1881—82. EDWARD LONG FOX, M.D. Oxon.
1882—83. JAMES GEORGE DAVEY, M.D. St. And.
1883—84. WILLIAM JOHNSTONE FYFFE, M.D., B.A. Dub.
1884—85. GEORGE FORSTER BURDER, M.D. Aberd.
1885—86. WILLIAM HENRY SPENCER, M.D., M.A. Cantab.
1886—87. FRANCIS POOLE LANSDOWN.
1887—88. LEMUEL MATTHEWS GRIFFITHS.
1888—89. ROBERT SHINGLETON SMITH, M.D., B.Sc. Lond.
1889—90. NELSON CONGREVE DOBSON, Ch.M. Brist.
1890—91. SAMUEL HENRY SWAYNE.
1891—92. FRANCIS RICHARDSON CROSS, M.B. Lond., LL.D. Brist.
1892—93. EDWARD MARKHAM SKERRITT, M.D., B.S., B.A. Lond.
1893—94. JAMES GREIG SMITH, M.B., C.M., M.A. Aberd., F.R.S.E.
1894—95. ALFRED JAMES HARRISON, M.B. Lond.
1895—96. ARTHUR WILLIAM PRICHARD.
1896—97. ALFRED EDWARD AUST LAWRENCE, M.D., C.M. Aberd.
1897—98. JOHN EDWARD SHAW, M.B., C.M. Ed.
1898—99. ROBERT ROXBURGH, M.B. Ed.
1899—00. WILLIAM HENRY HARSANT.
1900—01. DAVID SAMUEL DAVIES, M.D. Lond.
1901—02. BARCLAY JOSIAH BARON, M.B., C.M. Ed.
1902—03. GEORGE MUNRO SMITH, M.D. Brist.
1903—04. JAMES PAUL BUSH, C.M.G., C.B.E., Ch.M. Brist.
1904—05. REGINALD EAGER, M.D. Lond.
1905—06. JOHN DACRE.
1906—07. JAMES TAYLOR.
1907—08. HENRY WALDO, M.D., C.M. Aberd.
1908—09. JOHN MICHELL CLARKE, M.D., M.A. Cantab.
1909—10. JAMES SWAIN, C.B., C.B.E., M.D., M.S. Lond.
1910—11. BERTRAM MITFORD HERON ROGERS, M.D., B.Ch., B.A. Oxon.
1911—12. CHARLES ALEXANDER MORTON.
1912—13. WALTER CARLESS SWAYNE, M.D., B.S. Lond. and Brist.
1913—14. PATRICK WATSON-WILLIAMS, M.D. Lond., M.D. (Hon. Causa) Brist.
1914—15. WILLIAM HARRY CHRISTOPHER NEWNHAM, M.A., M.B. Cantab.
1915—16. GEORGE PARKER, M.A., M.D. Cantab., LL.D. Brist.
1916—17. FRANCIS HENRY EDGEWORTH, M.A., M.D. Cantab., D.Sc. Lond.
1917—18. FREDERICK LACE.
1918—19. ROBERT GUTHRIE POOLE LANSDOWN, M.D., B.S. Durh.
1919—20. I. WALKER HALL, M.D., Ch.B. Vict.
1920—21. LEOPOLD ERNEST VALENTINE EVERY-CLAYTON, M.D. Lond.
1921—22. CYRIL HUTCHINSON WALKER, B.A., M.B. Cantab.
1922—23. JOHN ALEXANDER NIXON, C.M.G., B.A., M.D. Cantab.
1923—24. JOHN LACY FIRTH, M.D., M.S. Lond.
1924—25. JOHN ODERY SYMES, M.D. Lond.
1925—26. THOMAS CARWARDINE, M.S. Lond.
1926—27. ARTHUR LAUNCELOT FLEMMING, M.B., Ch.B. Brist.
1927—28. WALTER KENNETH WILLS, M.A., M.B., B.Ch. Cantab.
1928—29. HENRY LAWRENCE ORMEROD, M.D., B.Ch. R.U.I.
1929—30. CHARLES FERRIER WALTERS.
1930—31. DAVID CHARLES RAYNER, Ch.M. Brist.
1931—32. JOHN ROGER CHARLES, M.A., M.D. Cantab.
1932—33. ERNEST WILLIAM HEY GROVES, M.D., M.S., D.Sc., B.Sc. Lond.
1933—34. HERBERT ELWIN HARRIS, B.A., M.B. Cantab.
1934—35. A. RENDLE SHORT, B.Sc., M.D. Lond.
1935—36. ARTHUR ERNEST ILES, O.B.E., M.B., B.S. Lond.
1936—37. RICHARD CHRISTOPHER CLARKE, O.B.E, M.B. Lond.
1937—38. CLIFFORD A. MOORE, M.B., M.S. Lond.
1938—40. LEONARD AUGUSTUS MOORE, M.B., Ch.B. Brist.
1940—45. CHARLES CORFIELD, M.D. Durh., Barr.-at-Law.
1945—46. H. H. CARLETON, M.A., M.D. Oxon., F.R.C.P. Lond.

1945.

### LIST OF SUBSCRIBERS

TO THE

### Bristol Medico-Chirurgical Journal.

### HONORARY MEMBER OF THE SOCIETY.

| | |
|---|---|
| SWAIN, JAMES, *C.B.*, *C.B.E.*, M.D., M.S. Lond. | Wyndham House, Easton-in-Gordano. |

### SUBSCRIBERS.

*Those marked * are Members of the Society.*

| | |
|---|---|
| *ADAMS, A. W., M.S. Lond. | Elton House, Clifton, Bristol 8. |
| ADAMS, J. B. | Kent Lodge, Eastbourne. |
| *ADAMS, S. B., Ph.D., M.B., Ch.B. Brist. | Clapton Hill, near Portishead. |
| *ALDWINCKLE, HELEN M., M.B., Ch.B. Brist. | 23 Rockland Road, Downend, Bristol. |
| *ALEXANDER, A. J. P., M.D. Belf. | Winsley Sanatorium, near Bath. |
| *ALEXANDER, D. A., M.B., Ch.B. Vict. | 112 Pembroke Road, Clifton Bristol 8. |
| ALEXANDER, G. L., B.A., M.B., B.Ch. Cantab. | West African Medical Service, Laura, via Accra, Gold Coast. |
| *ALFORD, A. F., M.B., Ch.B. Brist. | Ministry of Education, Belgrave Square, London, S.W.1. |
| ALFORD, H. J., M.D. Lond. | 4 Park Terrace, Taunton. |
| ASHTON, L. P., M.B., Ch.B. Brist. | Kapsowar, P.O. Eldoret, Kenya, East Africa. |
| *BAER, P., M.D. | |
| *BAIN, W. | 1 Greville Road, Southville, Bristol 3. |
| *BAKER, LILY A., M.D., B.Ch., B.A.O., R.U.I. | 23 Pembroke Road, Clifton, Bristol 8. |
| BALL, Mrs. J. J. | St. Leonard's Vicarage, Redfield, Bristol 5. |
| *BARBOUR, Lieut.-Col. R. F., R.A.M.C. | 13 Old Sneed Road, Stoke Bishop, Bristol. |
| *BAXTER, URSULA, M.B., Ch.B. Brist. | 167 Wells Road, Knowle, Bristol 4. |
| *BAXTER, V. T., M.B., Ch.B. | 167 Wells Road, Knowle, Bristol 4. |
| *BEATSON, D. H., M.B., Ch.B. Brist. | 8 Richmond Park Road, Clifton, Bristol 8 |
| *BECK, DIANA J. KINLOCH, M.B., B.S., F.R.C.S. | 7 Oakfield Road, Clifton, Bristol 8. |
| *BELL, R. J. IRVING | 5a Oakfield Road, Clifton, Bristol 8. |
| *BERGIN, F. G. | 31 Oakfield Road, Clifton, Bristol 8. |
| *BERRY, R. J. A., Prof., M.D., C.M. Edin. | 9 Derby Street, Ormskirk, Lancs. |
| *BIRRELL, J. A., M.D., B.S. Lond. | 1 Victoria Square, Bristol 8. |
| BLACKETT, J. F., M.D., B.S. Lond. | Hamsterley, Bloomfield Park, Bath. |
| BLETCHLY, G. PLAYNE, M.B. Lond. | c/o Lloyds Bank, Nailsworth, Glos. |
| *BODMAN, F. H., M.D., Ch.B. Brist. | 2 Redland Court Road, Bristol 6. |
| BOLT, R. F. | 70 Great Russell Street, London, W.C.1. |
| *BOULTON, B. J., M.B., Ch.B. Brist. | Ham Green Hospital, Pill, near Bristol. |
| *BOWN, NAOMI J., M.B., Ch.B. Brist. | The Corner, Nailsea, Somerset. |
| *BOYD, R. G., M.B., A.B., | Bristol General Hospital. |
| *BRADBEER, E. G., M.B., Ch.B. Brist. | 13 Percival Road, Bristol 8. |
| BRASHER, C. W. J., M.D. | Murivance, Nether Stowey, Somerset. |
| *BRINCKMAN, WINIFRED P., M.B., Ch.B. Brist. | 2 Clifton Park, Bristol 8. |
| *BRITTON, R. B., M.B., B.S. Lond. | 33a Whiteladies Road, Clifton, Bristol 8. |
| *BROCKLEHURST, R. J., M.A., D.M. Oxon. | The University, Bristol 8. |
| BROWN, J. E., M.B., Ch.B. Brist. | Coulthurst Villa, Brives Road, Maraval, Trinidad, B.W.I. |
| BRYAN, C. | 44 Nevil Road, Bishopston, Bristol 7. |
| *BRYANT, EUNICE, M.R.C.S., L.R.C.P. | 167 Redland Road, Bristol 6. |
| *BURGES, R. | Druid's Mead, Stoke Bishop, Bristol 9. |
| *BUSH, G. B., M.B., Ch.B. Brist. | Litfield House, Clifton, Bristol 8. |

| | |
|---|---|
| *CAIRNS, F. J. | Devon House, Whitehall Road, Bristol 5. |
| CAMERON, MARGARET, M.B., Ch.B. | 1 Dean Lane, Bristol 3. |
| *CAPPER, W. M., M.B., B.S. Lond. | Litfield House, Clifton, Bristol. |
| *CAREY, R. S., *O.B.E.*, B.A. Cantab. | 502 Bath Road, Brislington, Bristol 4. |
| *CARLETON, H. H., M.A., M.D., B.Ch. Oxon. | 1 Lansdown Road, Clifton, Bristol 8. |
| *CASSON, E., M.D., Ch.B. Brist. | Mount Pleasant, Clevedon. |
| *CHAMBERS, E. R. | 9 Clifton Park, Clifton, Bristol 8. |
| *CHARLES, J. R., M.A., M.D. Cantab. | Litfield House, Clifton Down, Bristol 8. |
| *CHITTY, H., M.S. Lond. | Naish House, Clapton-in-Gordano. |
| *CHRISTOFFERSON, P. E., M.B., Ch.B. Brist. | 14 Oakfield Road, Clifton, Bristol 8. |
| *CLAREMONT, L. E., M.D.S. Brist. | 5 Rodney Place, Clifton, Bristol 8. |
| *CLARK, Capt. DENNIS O., R.A.M.C. | |
| *CLARKE, R. C., *O.B.E.*, M.B., Ch.B. Brist. | Harley Lodge, Clifton Park, Bristol 8. |
| *COATES, H. | Notts County Mental Hospital, Radcliffe-on-Trent. |
| *COCHRANE, J. H. | 11 Bryant's Hill, St. George, Bristol 5. |
| *COLLINGWOOD, F. C., M.B., Ch.B. Brist. | 106 Foxley Lane, Purley, Surrey. |
| *COOKE, ELIAZBETH M., M.D. Lond., M.R.C.P. | 53 Queen's Court, Clifton 8. |
| *COOKE, R. V., Ch.M. Brist., F.R.C.S. | Litfield House, Bristol 8. |
| *COOKSON, R. G. | Clifton College, Bristol 8. |
| COOMBS, N. C. | Romaldkirk, Barnard Castle, Co. Durham. |
| *COOPER, W. R. | 13 Westfield Park, Redland, Bristol 6. |
| *CORFIELD, C., M.D. Durh. | 5 Kensington Place, Clifton, Bristol 8. |
| *CORNER, BERYL, M.D., B.S. Lond. | 3 Elton House, Rodney Place, Bristol 8. |
| *COSSHAM, W. L., M.B., Ch.B. Brist. | 3 Claremont Road, Bishopston, Bristol 7. |
| *COURTNEY, R. M., M.B., Ch.B. Glasg. | Manor House, Southmead Road, Bristol |
| *COXON, BEATRICE | 16 Richmond Hill, Clifton, Bristol 8. |
| *CRAIG, ANNE A., M.D., B.S. Lond. | 35 Queen's Court, Clifton, Bristol 8. |
| *CROOK, B. A., M.B., Ch.B. Brist. | The Laurels, Timsbury, near Bath. |
| CROOT, Lt.-Col. H. J., R.A.M.C. | Indian Gen. Hosp. (C), India Command. |
| *CROSSMAN, F. W., M.B. Durh. | White's Hill, Hambrook, near Bristol. |

| | |
|---|---|
| *DATTA, S. S., M.D. Brist. | Snowdon House, Fishponds, Bristol. |
| *DAVIES, E. D. D. | Standish House, Stonehouse, Glos. |
| *DAVIES, DR. J. N. P. | c/o Director Medical Services, Entebbe, Uganda, Africa. |
| *DELANEY, N., M.B., Ch.B. Manch. | The Meade, Bishopsworth, Somerset. |
| *DISNEY, M., M.D. Lond. | On Active Service. |
| *DIX, C. | 14 Mortimer Road, Clifton, Bristol 8 |
| *DIXON, MARY, M.B., Ch.B. | Bristol Maternity Hospital. |
| *DIXON, T. B. | 11 Pembroke Road, Bristol 8. |
| *DODD, GRANTHAM, M.B., B.S. | 42 Woodstock Road, Bristol 6. |
| *DUERDEN, J. R., M.B., Ch.B. Brist. | 4 Cecil Road, Bristol 8. |
| *DUNSTER, MARJORIE, M.D., Ch.M., M.R.C.O.G. | Bristol General Hospital (St. Monica's). |
| *DUTTON, HILDA R. | 405 Stapleton Road, Bristol 5. |

| | |
|---|---|
| *EGLINTON, J. H. C. | Street, Somerset. |
| ELLIOTT, F. P., M.B., C.M. Aberd. | 113 Grove Road, Walthamstow, London, E.17. |
| *ELLIOTT, W. H. A., M.B., B.S. Lond. | 29 Old Sneed Avenue, Stoke Bishop, Bristol 9. |
| *EMERY, J. L. | Childrens' Hospital, Bristol 2. |
| *EVANS, G. M., M.B., Ch.B. Brist. | 117 Ashley Road, Bristol. |
| *EYRE-BROOK, A. L., M.B., M.S. Lond. | The Cottage, Portishead. |

| | |
|---|---|
| *FELLS, G. R., M.B., Ch.B. Brist. | 19 Southmead Road, Bristol. |
| *FELLS, R. R., M.B., B.S. Lond. | 17 Mortimer Road, Clifton, Bristol 8. |
| *FENELEY, G. L., M.B., Ch.B. Brist. | Englewood, Falcondale Road, Westbury-on-Trym. |
| *FISHER, A. C., M.D. Brist. | Roan Antelope Mine, N. Rhodesia. |
| *FITZGIBBON, G. M., M.D. Lond. | 75 Pembroke Road, Clifton, Bristol 8. |

| | |
|---|---|
| *FORRESTER-BROWN, MAUD, M.D., M.S. Lond. | 22 Combe Park, Bath. |
| *FOSS, G. L., M.B., B.Ch. Cantab. .. .. .. .. | Cloud's Hill House, St. George, Bristol 5. |
| *FOX, F. E., B.A. Cantab. .. .. .. .. .. .. .. | Brislington House, Brislington, Bristol 4. |
| *FRASER, A. D., M.A., M.B., Ch.B. Glasg. .. | 4 Alexandra Road, Clifton, Bristol 8. |
| *FRASER, D., M.B., Ch.B. Edin. .. .. .. | Westgate Dursley, Glos. |
| *FULLER, H. L. .. .. .. .. .. .. .. .. .. | 9 Gay Street, Bath. |
| | |
| *GARDEN, R. R., M.A., M.B., B.Ch. Aberd. .. | 9 Harley Place, Clifton, Bristol 8. |
| GARLAND, E. B., M.B., C.M. Edin. .. .. .. .. | 114a Hampton Road, Redland, Bristol 6. |
| *GERRISH, NORMAN, M.B., Ch.B. Brist. .. .. | 2 Station Road, Keynsham, Som. |
| *GIBBS, J. R. .. .. .. .. .. .. .. .. .. .. .. | 48 Codrington Road, Bishopston, Bristol 7. |
| *GOLDING, H. M., *D.F.C.*, M.B., Ch.B. .. .. | 10 Barton Hill Road, Bristol 5. |
| *GORHAM, A. P., M.B., Ch.B. Brist. .. .. .. | 53 Westbury Road, Westbury-on-Trym. |
| *GORNALL, W. A., M.D. Brist. .. .. .. .. .. | Ulvik, Nailsea, near Bristol. |
| GRIFFITHS, CORNELIUS A. .. .. .. .. .. .. | 35 Newport Road, Cardiff. |
| | |
| *HAM, C. I., M.B., Ch.B.Brist. .. .. .. .. .. | 89 Stonebridge Park, Eastville, Bristol. |
| *HARRIS, H. E., M.A., M.B., B.Ch. Cantab. .. | 13 Lansdown Place, Clifton, Bristol 8. |
| *HAWKINS, MONICA, M.B., Ch.B. .. .. .. | Cowslip Green, Wrington. |
| *HEALES, J. N., M.B., Ch.B. Brist. .. .. .. | Holmcroft, Street, Somerset. |
| *HECTOR, F., M.D. Lond. .. .. .. .. .. .. | 1 Victoria Square, Clifton, Bristol 8. |
| *HEMPHILL, R., M.D. Dub. .. .. .. .. .. | Clonora, Blackberry Hill, Fishponds. |
| *HENDERSON, JAMES, M.B., Ch.B. Aberd. .. | The City Sanatorium, Yardley Road, Birmingham. |
| *HERAPATH, C. E. K., *M.C.*, M.D. Lond. .. | Park Close, 92 Charlton Road, Keynsham. |
| *HEWER, Professor T. F., M.D. Brist. .. .. | Canynge Hall, Bristol 8. |
| *HILL L. C., M.D. .. .. .. .. .. .. .. .. | 11 The Circus, Bath. |
| *HILL, WINIFRED, M.B., Ch.B. Brist. .. .. | c/o Lloyd's Bank, Ilford. |
| *HOFFMAN, H. LOVELL, M.D., B.Ch. Cantab. .. .. | 26 Circus, Bath. |
| *HOLLAND, W. A. L. .. .. .. .. .. .. .. .. | 1 Upper Belgrave Road. Clifton. |
| *HOOKER, J. A., Colonel, M.B., Ch.B.Brist. .. | 16 St. Edyth's Road, Sea Mills 9. |
| *HOSKEN, J. G. F... .. .. .. .. .. .. .. .. | Uley, Glos. |
| HOSPITAL, BRISTOL GENERAL | |
| *HOUGHTON, LYDIA M., M.B., B.S. Lond. .. | 74 Church Road, Redfield, Bristol 5. |
| *HOWELL, T. E. .. .. .. .. .. .. .. .. .. .. | 6 Richmond Hill, Bristol 8. |
| *HUGHES, F. W. TERRELL, M.B., Ch.B. Brist. | Chew Magna, Somerset. |
| *HUGHES, MARGUERITE G., M.B., Ch.B. Brist. | 11A Miles Road, Clifton. |
| *HUNTER, F. S. .. .. .. .. .. .. .. .. .. | Penrose Cottage, Clifton Down, Bristol 8. |
| *HYATT, ANNIE W., M.B., B.S. Lond. .. .. | Merrymead, Shepton Mallet, Som. |
| | |
| *ILES, ANNA, M.B., Ch.B. Brist. .. .. .. .. | 87 Pembroke Road, Clifton, Bristol 8. |
| *ILES, A. E., *OB.E.*, M.B., B.S. Lond. .. .. | 87 Pembroke Road, Clifton, Bristol 8. |
| INFIRMARY, BRISTOL ROYAL | |
| INGLE, C. DURBAN .. .. .. .. .. .. .. | Somerton, Somerset. |
| *ISSERLIN, B. .. .. .. .. .. .. .. .. .. .. | 21 St. John's Road, |
| | |
| *JACKMAN, W. A., *T.D.*, M.B., Ch.B. Brist. .. | 15 Mortimer Road, Clifton, Bristol 8. |
| *JAMES, J. A., M.D. Lond. .. .. .. .. .. .. | Litfield House, Clifton Down. Bristol 8. |
| *JAMES, J. A., Senr. .. .. .. .. .. .. .. .. | Dusk, Elmlea Avenue, Stoke Bishop, Bristol, 9. |
| *JENKINS, F. G., M.B., Ch.B. Brist. .. .. .. | 51 Redcliff Hill, Bristol 1. |
| *JENKINS, R. D. .. .. .. .. .. .. .. .. .. .. | The Copper Beeches, Almondsbury, near Bristol. |
| *JENKINS, P. K., *M.C.*, M.B., Ch.B. .. .. .. | R.A.M.C. |
| JOHNSON-MASON, A. J. .. .. .. .. .. .. | Brentry House, Brentry. |
| *JOHNSON, R. G., M.B. Lond. .. .. .. .. .. | 119 Redland Road, Bristol 6. |
| *JONES, MEGAN E. .. .. .. .. .. .. .. | Royal Infirmary, Bristol 2. |

| | |
|---|---|
| *KEMM, N. F., M.B., B.S. Lond. .. .. .. | 9 Miles Road, Clifton, Bristol 8. |
| *KERSLEY, G. D., M.D. .. .. .. .. .. .. | 6 The Circus, Bath. |
| *KINDERSLEY, M.C., Ch.B., B.A. Cantab. .. | 18 The Circus, Bath. |
| KING, E. F. .. .. .. .. .. .. .. .. .. .. | 2 Upper Wimpole Street, London, W.1. |
| *KINGSTON, S. H., M.B., Ch.B. Brist. .. .. | 3 Whiteladies Road, Clifton, Bristol 8. |
| *KYLE, H. G., M.A., M.D., B.Ch. Oxon. .. .. | 31 Westbury Road, Bristol. |
| | |
| *LACE, F .. .. .. .. .. .. .. .. .. .. | 5 Gay Street, Bath. |
| LALONDE, T. P., M.B., Ch.B. Brist... .. .. | Wykeham House, Romsey, Hants. |
| *LEE-MICHELL, R., M.R.C.S., L.R.C.P. .. .. | Clifton Lodge, Cheddon Road, Taunton. |
| *LEES, E. LEONARD, M.D., C.M. Edin. .. .. | 28 Downs Park West, Bristol 6. |
| *LEWIS, F. J., M.B., Ch.B. Brist. .. .. .. | Canynge Hall, Bristol 8. |
| *LOGAN, H. B. .. .. .. .. .. .. .. .. | Elmwood, Aberdeen Road, Cotham Bristol 6. |
| *LOXTON, S. D., M.B., Ch.B. .. .. .. .. .. | |
| *LUCAS, J E. .. .. .. .. .. .. .. .. .. | c/o 132 York Road, Bristol 3. |
| *LUCAS, J J. S., M.D. Lond. .. .. .. .. | 186 Wells Road, Knowle, Bristol 4. |
| *LUCAS, J V... .. .. .. .. .. .. .. .. .. | 186 Wells Road, Knowle, Bristol 4. |
| *LUCAS R. P., M.B., Ch.B. Brist. .. .. .. | Northways, Fallodon Way, Henleaze. |
| | |
| *MCKEN E, W. G., *M.C.* .. .. .. .. .. .. | 11 Bath Road, Reading. |
| *MACLACHLAN, A. M., M.B., Ch.B. Glasg. .. | 65 Redcliff Hill, Bristol 1. |
| *MARSHALL, A. H., M.B., Ch.B. Brist. .. .. | 281 Canford Lane, Bristol. |
| *MARWOOD, S. F., M.D., B.S. .. .. .. .. | Troy House, Kingswood, Bristol. |
| *MACLAREN, CATHERINE J., M.B., Ch.B. Edin. | Hereford House, Clifton Down. |
| MACLEAN, SIR EWEN J., M.D., C.M. Edin. .. | 12 Park Place, Cardiff |
| *MACLEOD, G., M.A. Glas., M.D., M.B. .. .. | Wargrave, Clevedon, Somerset. |
| MARSHALL, A. L., M.B., B.A. Cantab. .. .. | Laxfield, Woodbridge, Suffolk. |
| *MARTIN, P. S., *M.C.* .. .. .. .. .. .. .. | Victoria Quadrant, Weston-super-Mare. |
| MASON, J. JOHNSON .. .. .. .. .. .. .. | Brentry House, Brentry. |
| *MATHESON, N. R., M.B., Ch.B.. .. .. .. | 37 Abbey Road, Westbury-on-Trym. |
| MIALL, C. L'O .. .. .. .. .. .. .. .. .. | Elm Hayes, Paulton, near Bristol. |
| *MILLING, T., M.B., Ch.B. Belf. .. .. .. .. | 1 Vicarage Road, Southville, Bristol 3. |
| *MOHAN, S. L., M.B., B.S... .. .. .. .. | 225 Stapleton Road, Bristol 5. |
| *MOORE, C. A., M.B., M.S. Lond. .. .. .. | South Lodge, Kingsweston Road, Henbury, Bristol. |
| *MOORE, L. A., M.B., Ch.B. Brist. .. .. .. | Hillsborough, Cotham Park, Bristol 6. |
| *MOORE, R. H., M.B., Ch.B. Brist. .. .. .. | White Cross Court, Wells Road, Bristol 4. |
| *MUNDY, G. S., M.B., B.Ch. Brist. .. .. .. | Homewood, Coombe Dingle, Bristol. |
| *MURPHY, F. D., M.B. .. .. .. .. .. .. | 20 Gay Street, Bath. |
| *MYLES, Q. St. L., M.A., B.M., B.Ch. Oxon. .. | 7 Apsley Road, Clifton, Bristol 8. |
| | |
| *NICHOLSON-LAILEY, J. R. .. .. .. .. .. .. | Elmfield House, South Road, Taunton. |
| *NIXON, DOREEN, M.B., B.S. Lond. .. .. .. | 7 Lansdown Place, Clifton, Bristol 8. |
| *NIXON, J. A., *C.M.G.*, M.D. Cantab. .. .. | 7 Lansdown Place, Clifton, Bristol 8. |
| *NOBLE, J. I., M.B., Ch.B. Liverp. .. .. .. | 102 Pembroke Road, Clifton, Bristol 8. |
| *NORMAN, R. M., M.D. Brist. .. .. .. .. | Highwood, Stapleton Park, Bristol. |
| | |
| O'DONOHOE, MONICA A., M.B., Ch.B. .. .. | 1 Beaconsfield Road, Bristol 8. |
| ORMEROD, G. L. M.A., M.B., B.Ch. Cantab .. | 2 Henleaze Road, Westbury-on-Trym, Bristol. |
| *ORR-EWING, H. J., *M.C.*, M.D. Lond. .. .. | 1 Beaufort Buildings, Clifton, Bristol 8. |
| | |
| *PALIN, A. G., B.M., B.Ch. Oxon. .. .. .. | Litfield House, Clifton Down, Bristol 8. |
| *PARKES, J. A., M.B., Ch.B. Liverp. .. .. | 8 South Road, Redland, Bristol 6. |
| *PARKES, P. W. J. .. .. .. .. .. .. .. | 164 Filton Avenue, Bristol 7. |
| PARKINSON, Lt.-Col. G. S., *D.S.O.*, R.A.M.C. | London School of Hygiene and Tropical Medicine, Keppel Street, London, W.C. 1. |

| | |
|---|---|
| *PARRY, R. H., M.B., B.S.Lond. | Public Health Offices, Bristol 1. |
| PARSONS, Sir J. H., M.B., B.S., B.Sc. Lond. | 54 Queen Anne Street, London, W. |
| *PAUL, R. G. | 23 Pembroke Road, Bristol 8. |
| *PAULI, C. H. | Pagan's Hill Farm, Chew Stoke. |
| *PERRY, B., M.D., Ch.B.Brist. | Canynge Hall, Clifton, Bristol 8. |
| PHELPS, P. C., M.B., Ch.B. | 23 St. Alban's Road, Bristol 6. |
| *PHILLIPS, P., M.B., Ch.B. Brist. | Southmead Hospital, Bristol. |
| *PIRRIE, A. L., M.B., Ch.B. Glasg. | 528 Kensington Hill, Bristol. |
| *POCOCK, J. A., M.A., M.B., B.Ch. Cantab. | 75 Drayton Gardens, London, S.W. 10. |
| PORTER, C. P. | 28 Mill Street, Kidderminster. |
| *PORTER CHARLES, M.A. Oxon. | 124a Redland Road, Bristol 6. |
| *POTTER, MABEL F., M.D., Brist. | 19 Royal Park, Clifton, Bristol 8. |
| *POWELL, H. F., M.D. Brux. | Ellenborough Park, Weston-super-Mare. |
| *PRATT, MAJOR W. W., D.T.M.V.H. | Feltham Farm, Pucklechurch. |
| *PRICE, ANNE B., B.Sc., M.B., Ch.B. | 7 Stoke Lane, Stapleton. |
| *PRICE, C. H. G., M.B., Ch.B. Brist. | Claverham House, Claverham, Yatton. |
| *PRIDIE, K. H., M.B., B.S. Lond. | 58 St. John's Road, Clifton, Bristol 8. |
| *PRIDIE, KENNETH, Mrs. | 58 St. Johns Road, Bristol 8. |
| *PROWSE, D. C., M.B., Ch.B. Brist. | Wigmore House, Thornbury, near Bristol. |

| | |
|---|---|
| *RANKIN, P. C., M.B., Ch.B. Glasg. | Lawrence Hill House, Bristol. |
| *RIDGWAY, J., M.B., Ch.B. | Hanham, Bristol. |
| *ROBERTS, J. A. F., M.B., Ch.B. Edin. | Stoke Park Colony, Bristol. |
| *ROBERTS, J. A. L., M.B., Ch.B. | 75 High Street, Kingswood, Bristol. |
| *ROBERTSON, D., M.B., Ch.B. Edin. | 2 Knowle Road, Knowle, Bristol 4. |
| *ROBERTSON, L. G., M.B., Ch.B. | 162 Redland Road, Bristol 6. |
| *ROGERS, H., M.D. Brist. | 91 Old Park Ridings, Grange Park, N.21. |
| *ROLFE, R., B.A., M.B., B.C. Cantab. | Park House, St. Andrew's Road, Avonmouth. |
| *ROWLANDS, R. D. | Connaught Military Hospital, Knaphill, Surrey. |
| *ROWLEY, A. T. | 13 Walliscote Road, Weston-super-Mare. |
| *RUDOLF, MAJOR G. DE M. | Seawalls, Clevedon. |
| *RYAN, P. B., M.B., Ch.B. | The Paddock, Frenchay. |

| | |
|---|---|
| *SAMPSON, O. E. L., M.B., Ch.B. Brist. | 77 Stackpool Road, Bristol 3. |
| *SCARFF, G., M.B., Ch.B. Edin. | 83 Pembroke Road, Clifton, Bristol 8. |
| *SHEPHERD, H. L., M.B., Ch.M. Brist. | 79 Pembroke Road, Clifton, Bristol 8. |
| *SHORE, T. L. H., M.A., M.B., B.Ch. | Linden Lodge, Holmes Hill, Taunton. |
| *SHORT, A. R., F.R.C.S., M.D. Lond. | 69 Pembroke Road, Clifton, Bristol 8. |
| *SIMPSON, T. E, N. | "West Hythe," Old Patchway, Bristol. |
| *SMYTHE, H. J. D., *M.C.*, M.D., M.S. Lond. | On Active Service. |
| *SNAWDON, J. W. E., M.B., Ch.B., B.D.S. | Litfield House, Clifton, Bristol 8. |
| *SOLTAU, H. K. V., M.D. Lond. | 19 The Avenue, Clifton, Bristol 8. |
| *SOMERS, MARY A. E. | 2 Ashcombe Road, Weston-super-Mare. |
| *SPARKS, J. V., | The Middle House, Felton. |
| *SPOOR, A., M.B., Ch.B. Cantab. | The Hydro, Bristol 1. |
| *STEPHEN, R. J. | 92 Redland Road, Bristol 6. |
| *STROVER, H. W. M., *O.B.E.*, M.B., Ch.B. Aberd. | 90 Redland Road, Bristol 6. |
| *STRUTHERS, A. J., M.B., Ch.B. Glasg. | 100 Church Road, Redfield, Bristol 5. |
| STRUTHERS, GEO., M.B., Ch.B. Glasgow | 100 Church Road, Bristol 5. |
| *SUTTON, G. E. F., M.D. Lond. | 1 Pembroke Road, Clifton, Bristol 8. |
| *SYMES, J. O., M.D. Lond. | 6 Pembroke Vale, Clifton, Bristol 8. |

| | |
|---|---|
| *TALLACK, MAJOR R. J. K., R.A.M.C. | (Kenya) Field Amb., A.P.O., E.A. Command. |
| *TASKER, D. G. C., M.B., M.S. Lond. | 9 College Fields, Clifton, Bristol 8. |
| *TAYLOR, A. L., M.D. Lond. | The General Hospital, Bristol 1. |

| | |
|---|---|
| TAYLOR, A. L. .. .. .. .. .. .. .. .. | The Royal Infirmary Bristol 2. |
| THIN, J. .. .. .. .. .. .. .. .. .. .. | 54 and 55 South Bridge, Edinburgh. |
| *TODD, A. T., *O.B.E.*, M.D. Edin. .. .. .. .. | Royal Park House, Clifton, Bristol 8. |
| *TRIBE, DR. M. N., M.B., Ch.B. .. .. .. | Ding's House, St. Philips, Bristol 2. |
| TRINICK, RICHARD H. .. .. .. .. .. .. .. | 42 Cherry Valley Gardens, Belfast. |
| TRIPP, DOROTHY M. H. .. .. .. .. .. .. | Mission Hospital, Karim Nagar, Nizam's Dominions, South India. |
| *TRYON, VICTORIA S., M.B., Ch.B. Brist. .. | 3 Harley Place, Clifton, Bristol 8 |
| | |
| *WAGSTAFFE, E. M., M.B., Ch.B. .. .. .. | Winford Orthopædic Hospital. |
| *WANSBROUGH, T. B., M.B., Ch.B. Brist. .. | 23 Pembroke Road, Clifton, Bristol 8. |
| *WALKER, CYRIL H., B.A., M.B. Cantab. .. | Harewood, Clapton-in-Gordano, Som. |
| WAKE, S. C., M.B., Ch.B. Brist. .. .. .. | "Stanhope," Bideford, N. Devon. |
| *WARD, H. W. .. .. .. .. .. .. .. .. .. | Chipping Manor, Wotton-under-Edge. |
| *WARREN, R., M.A., M.D. Oxon. .. .. .. | Heathgates, Weston-super-Mare. |
| *WATSON-WILLIAMS, E., *M.C.*, B.A., M.B. Cantab., Ch.M. Brist. | 65 Pembroke Road, Clifton, Bristol 8. |
| WATTS, J. B. V. .. .. .. .. .. .. .. .. | Frantzina's Rust, Barberton, Transvaal, S. Africa. |
| WEDDELL, C. W. .. .. .. .. .. .. .. .. | Boydville, 46 Miller Street, West Melbourne, Australia. |
| *WEEKS, A., L.M.S.S.A. .. .. .. .. .. .. | 39 Badminton Road, Downend. |
| *WHITE, R. F., M.B., Ch.B. .. .. .. .. | "Wellclose," High Street, Nailsea. |
| *WIGODER, SYLVIA, M.A., M.D., Ph.D. .. .. | |
| *WILBOND, R. G. .. .. .. .. .. .. .. .. | 76 Chesterfield Road, St. Andrew's Pk., Bristol. |
| *WILLIAMS, I., M.B., Ch.B. Brist. .. .. .. | "Ardrem," Hill View, Henleaze, Bristol. |
| *WILLIAMS, S. R., M.B. Lond. .. .. .. .. | Buckfastleigh, Devon. |
| *WILLOUGHBY, J., M.B. Ch.B. .. .. .. .. | "Kingsholme," Clevedon Road, Nailsea. |
| *WOOD, D. .. .. .. .. .. .. .. .. .. .. | 105 Pembroke Road, Clifton, Bristol 8. |
| *WOOD, URSULA, M.B., Ch.B. .. .. .. .. | Henley Lodge, Yatton, Som. |
| *WOOD, W. V., *M.C.*, B.A. Cantab. .. .. .. | Henley Lodge, Yatton, Som. |
| *WOODMAN, DOROTHY, M.D. Lond. .. .. .. | 10 Tyndall's Park Road, Clifton, Bristol 8. |
| *WOOLLEY, W., M.B., Ch.B. Brist. .. .. .. | 239 Cranbrook Road, Redland. |
| *WRIGHT, A. J. M., M.B., B.S. Lond. .. .. | Harley Cottage, Clifton Park, Clifton, Bristol 8. |
| *WRIGHT, WINIFRED M., M.B., B.S. Lond. .. | Merrymead, Shepton Mallet, Som. |
| | |
| *ZEALAND, L., M.D. Man. .. .. .. .. .. .. | Brecon Lodge, Westbury-on-Trym, Bristol. |

### Index

Anniversary (75th) of *The Practitioner*, 32

Book Reviews, 27
Bristol University.—
Medical Library, 37–40
Examination Results, 41

Churchill, Mr. Winston, and Standish House, 32

Daniel, G. H.—Social and Economic Conditions and the Incidence of Rheumatic Heart Disease, 13

Elcock, Charles E.—The Future of Health and Hospital Services from an Architect's Point of View, 1

F.R.C.S.—New Regulations, 32
Future of Health and Hospital Services from an Architect's Point of View. —Charles E. Elcock, 1

New Regulations for the F.R.C.S., 32

Obituaries.—
F. H. Edgeworth, 33
J. L. Firth, 36
R. H. Tasker, 35

*Practitioner, The*.—Seventy-fifth anniversary, 32

Social and Economic Conditions and the Incidence of Rheumatic Heart Disease.—G. H. Daniel, 13
Standish House and Mr. Winston Churchill, 32

### Index to Vol. LXII.

Acute Inversion of the Uterus following delivery.—C. Rendle Short, 9

Appointments.—
Sir Lionel Whitby, 28
Surg. Rear-Admiral H. St. C. Colson, 29
Professor R. Milnes Walker, 29

Bodman, F.—Selection of Medical Students, 18

Examination Results, 42

Gastroscopy.—N. C. Tanner, 1

Hospital Survey of the South-West, 29

Library, 39

Medical Students, Selection of.—F. Bodman, 18

Nierenstein, M.—Rock Carvings of Surgical Instruments in East Africa, 14

Obituaries.—
John Dacre, 31
E. W. Hey Groves, 31
C. A. Joll, 34
L. N. Morris, 35
N. L. Price, 35
D. C. Rayner, 36
C. F. Walters, 35

Rendle Short, C.—Acute Inversion of the Uterus following Delivery, 9

Reviews of Books, 22

Rock Carvings of Surgical Instruments in East Africa.—M. Nierenstein, 14

Selection of Medical Students.—F. Bodman, 18

Surgical Instruments, Rock Carvings of, in East Africa.—M. Nierenstein, 14

Tanner, N. C.—Gastroscopy, 1

Uterus, Acute Inversion of, following Delivery.—C. Rendle Short, 9

Victory in Europe, 28

